# CerealESTDb: A Comprehensive Resource for Abiotic Stress-Responsive Annotated ESTs With Predicted Genes, Gene Ontology, and Metabolic Pathways in Major Cereal Crops

**DOI:** 10.3389/fgene.2022.842868

**Published:** 2022-02-24

**Authors:** Sanjeev Kumar, Jyotika Bhati, Arijit Saha, Shashi Bhushan Lal, Pankaj Kumar Pandey, Dwijesh Chandra Mishra, Mohammad Samir Farooqi, Anuj Kumar, Krishna Kumar Chaturvedi, Anil Rai

**Affiliations:** Centre for Agricultural Bioinformatics, ICAR-Indian Agricultural Statistics Research Institute, PUSA, New Delhi, India

**Keywords:** cereals, abiotic stress, ests, genes, pathways, ontology

## Abstract

Cereals are the most important food crops and are considered key contributors to global food security. Loss due to abiotic stresses in cereal crops is limiting potential productivity in a significant manner. The primary reasons for abiotic stresses are abrupt temperature, variable rainfall, and declining nutrient status of the soil. Varietal development is the key to sustaining productivity under influence of multiple abiotic stresses and must be studied in context with genomics and molecular breeding. Recently, advances in a plethora of Next Generation Sequencing (NGS) based methods have accelerated the enormous genomic data generation associated with stress-induced transcripts such as microarray, RNAseq, Expressed Sequenced Tag (ESTs), etc. Many databases related to microarray and RNA-seq based transcripts have been developed and profusely utilized. However, an abundant amount of transcripts related to abiotic stresses in various cereal crops arising from EST technology are available but still remain underutilized in absence of a consolidated database. In this study, an attempt has been made with a primary goal to integrate, analyse, and characterise the available resources of ESTs responsive to abiotic stresses in major cereals. The developed CerealESTdb presents a customisable search in two different ways in the form of searchable content for easy access and potential use. This database comprises ESTs from four major cereal crops, namely rice (*Oryza sativa* L.), wheat (*Triticum aestivum* L.), sorghum (*Sorghum bicolour* L.), and maize (*Zea mays* L.), under a set of abiotic stresses. The current statistics of this cohesive database consists of 55,826 assembled EST sequences, 51,791 predicted genes models, and their 254,609 gene ontology terms including extensive information on 1,746 associated metabolic pathways. We anticipate that developed CerealESTdb will be helpful in deciphering the knowledge of complex biological phenomena under abiotic stresses to accelerate the molecular breeding programs towards the development of crop cultivars resilient to abiotic stresses. The CerealESTdb is publically available with the URL http://cabgrid.res.in/CerealESTDb.

## Introduction

To provide more than nine billion people by 2050 with essential nutrients and energy in their everyday diet, food production must be increased by 70% with reduced fertilizer inputs (Wani et al., 2021; Kumar et al., 2018; Balyan et al., 2016). Crop productivity suffers due to multiple abiotic stresses during the crop life cycle. The important four cereal crops, namely rice (*Oryza sativa* L.), wheat (*Triticum aestivum* L.), sorghum (*Sorghum bicolour* L.), and maize (*Zea mays* L.) show sensitivity to a wide range of abiotic stress viz*.,* abscisic acid (ABA), cold, drought, heat, and salt (Askari-Khorasgani et al., 2021; Cramer et al., 2011). In crop plants, mitigation mechanisms against these environmental stresses are complex biological processes. Although, there is a surge of biological information on plant responses to abiotic stresses, the understanding of the molecular mechanisms involved is still enigmatic (Zhang et al., 2021; Wani et al., 2018; Gahlaut et al., 2016). The mining of candidate genes and gene families responsive to multiple abiotic stresses from whole-genome sequencing data facilitates the molecular understanding of the complex cellular and molecular processes in a significant manner. Also, functional and structural annotations of genes and gene families will provide insight into the complex interactions regulating stress responses (Takahashi et al., 2020; Atkinson et al., 2013). Recently, advances in high throughput omics technologies have accelerated the identification and characterization of novel genes and their biological functions in a tissue-specific manner. At the genome and transcriptome level, the pathway reconstruction will play a principal role in quantifying and characterising the genotype to phenotype relationship (Gupta et al., 2020; Zhang et al., 2020; Feist and Palsson, 2008). In this ongoing genomic era, a huge amount of genomic data have been generated and analysed to provide key information leading to the development of crop cultivars resilient to abiotic stresses (Alter et al., 2015). This includes various transcriptomic data of gene expression such as Expressed Sequence Tags (EST), microarray, RNAseq etc. (Vincent et al., 2013; Zeng and Extavour, 2012). Whlie the era of Next Generation Sequencing (NGS) generates a huge amount of transcriptome data, previously generated EST resources still hold an important position. EST sequence has been defined as a short genomic sub-sequence and is important for the identification of gene transcripts under different environmental conditions. It can provide a means of accessing the gene space of almost any organism (Bouck and Vision, 2007; Nagaraj et al., 2007). EST libraries are a cost-effective tool and can serve as a starting point for the development of effective molecular genetic markers, such as gene-linked microsatellites, single nucleotide polymorphisms, etc. However, these EST resources are too scattered to provide insight into the biological process including the involvement of genes/regulatory elements and their functional role in responding to different abiotic stresses. Though the process of generation of EST data is historical, simple, cost-effective, and abundantly available it largely remains underutilised due to the scarce availability of proper databases. Efforts were made in the past by several research groups related to the development of EST resources in cereals such as Gramene (Jaiswal, 2011), cerealsDB (Wilkinson et al., 2012), Wheat Genome (Lai et al., 2012), MaizeGDB (Lawrence et al., 2004), Plant Stress Gene Database (Prabha et al., 2011), and Uniprot (Apweiler et al., 2004) summarized in Table 1.

**TABLE 1:** Summary of the comparison with other database resources.

Database	Developed	Updated	Web address	Advantages	Limitations
**Gramene** (Jaiswal, 2011)	2010	2019	http://www.gramene.org/	It is a curated, open-source, integrated data resource for comparative functional genomics in crops and model plant species	The information flow in this database is not very user-friendly as this is a generic data resource. End-users will have to carry out a rigorous search to extracting the required information
**cerealsDB** (Wilkinson et al., 2012)	2012	2020	https://www.cerealsdb.uk.net/cerealgenomics/CerealsDB/indexNEW.php	This database contains data related to all SNPs including flanking sequences as well as links to various other genomics tools for further analysis	This database contains SNP markers related to wheat crops only
**Wheat Genome** (Lai et al., 2012)	2012	No latest update	http://www.wheatgenome.info/	This repository stores Wheat genome sequences generated from different assembly processes	Information only on the genomic sequences fails to provide other useful information such as genes, proteins, pathwaysetc.
**MaizeGDB** (Lawrence et al., 2004)	2004	2020	http://www.maizegdb.org/	This service provides basic cultivation-related information on Maize crops. It also includes SNP, mutants, phenotypes, and other useful tools	This resource is lacking in providing the information related to genes, proteins, and pathways along with ontologies related to maize genomes
**Plant Stress Gene Database** (Prabha et al., 2011)	2011	No latest update	http://ccbb.jnu.ac.in/stressgenes/frontpage.html	This is a static database with 259 stress-related genes of 11 important species and these genes are linked to NCBI resources for further information	The limitation of this resource is in providing the proteins, pathways, and GO terms with respect to stress-specific genes
**Uniprot** (Apweiler et al., 2004)	2002	2020	https://www.uniprot.org/	This resource acts as an encyclopaedia of proteins and their related information	The stress-specific information for important cereal crops is not included in this data resource

Therefore, an attempt has been made to develop a user friendly, searchable, and interactive database of assembled and annotated ESTs for major cereal crops i.e., maize, rice, sorghum, and wheat along with associated genes and metabolic pathways involved in abiotic stresses *viz.*, ABA, cold, drought, heat and salt. In order to develop this database, these abiotic stresses related ESTs of considered cereal crops were collected from various sources and assembled. Subsequently, genes were identified and putative genes were predicted and further, these genes were functionally annotated using bioinformatics tools (Bhati et al., 2016; Chaduvula et al., 2015; Bhati et al., 2014). Partial amplification of nine identified candidate genes related to salinity stress response genes was standardised and carried out in *Oryza sativa* var. *indica* IR64 (Bhati et al., 2016), and 12 candidate genes were selected for salinity stress and amplified in nine indigenous *Sorghum bicolor* genotypes (Chaduvula et al., 2015). Moreover, information associated with various metabolic pathways involving these genes was identified and collected. This extracted and derived information was stored in relational database management systems (RDBMS) and made available through a searchable web interface as CerealESTdb which is unique in terms of its contents and features. This database provides various search options to retrieve the desired information in a few clicks and selections. This database contains 51,791 genes, 254,609 gene ontology terms, and 1,746 pathways. The primary goal of this database is to serve as a central data repository of processed and annotated EST resources of major cereal crops under abiotic stress conditions. The database is accessible from http://cabgrid.res.in/CerealESTDb.

## Materials and Methods

### Source of Data and Functional Annotation

**FIGURE 1:**
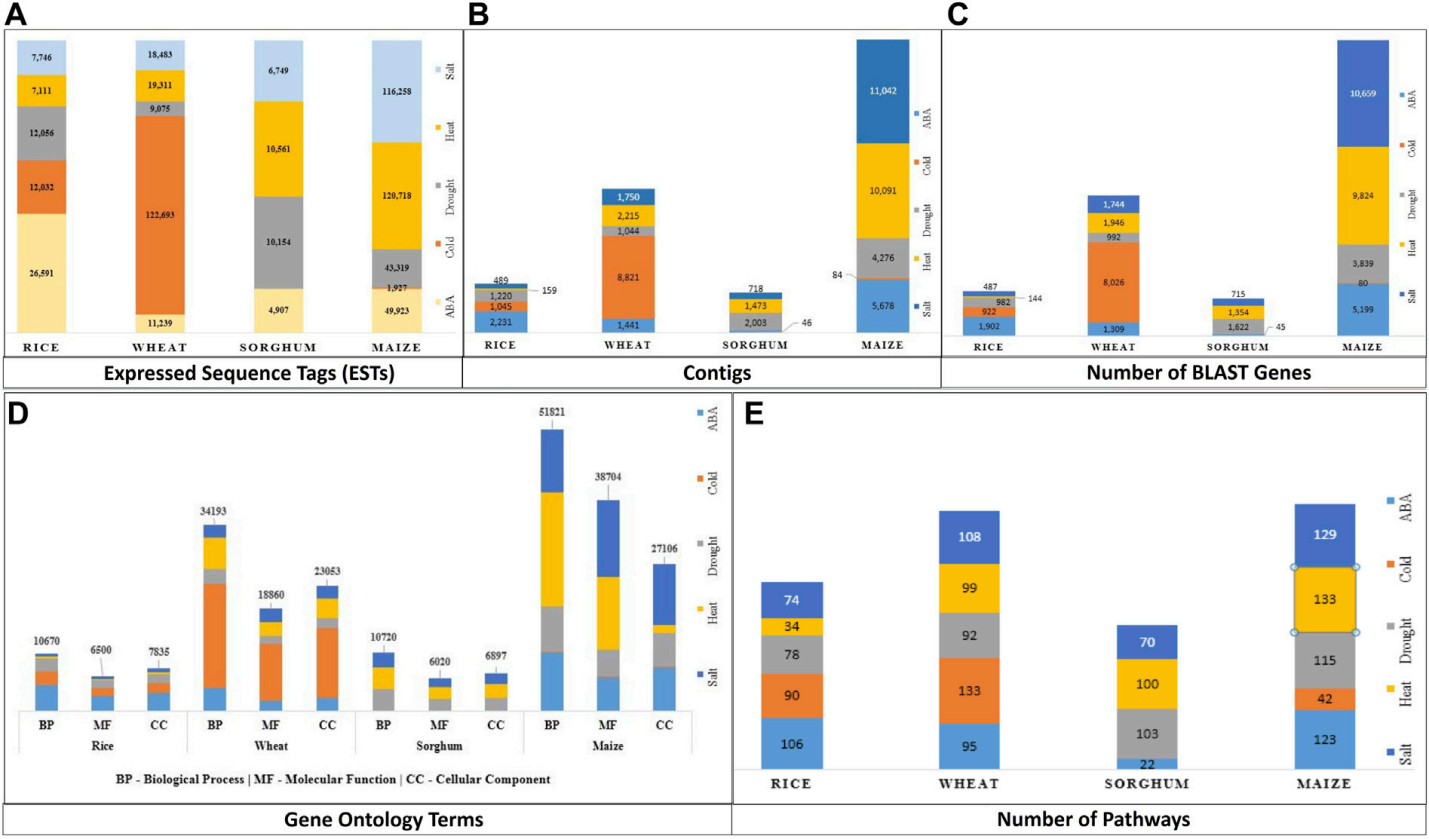
Data Summary—**(A)** ESTs, **(B)** Annotated contigs along with their corresponding **(C)** BLAST genes, **(D)** GO term, and **(E)** Pathway (GO: Gene Ontology, BP, Biological process; MF, Molecular Function; CC, Cellular Components).

**FIGURE 2:**
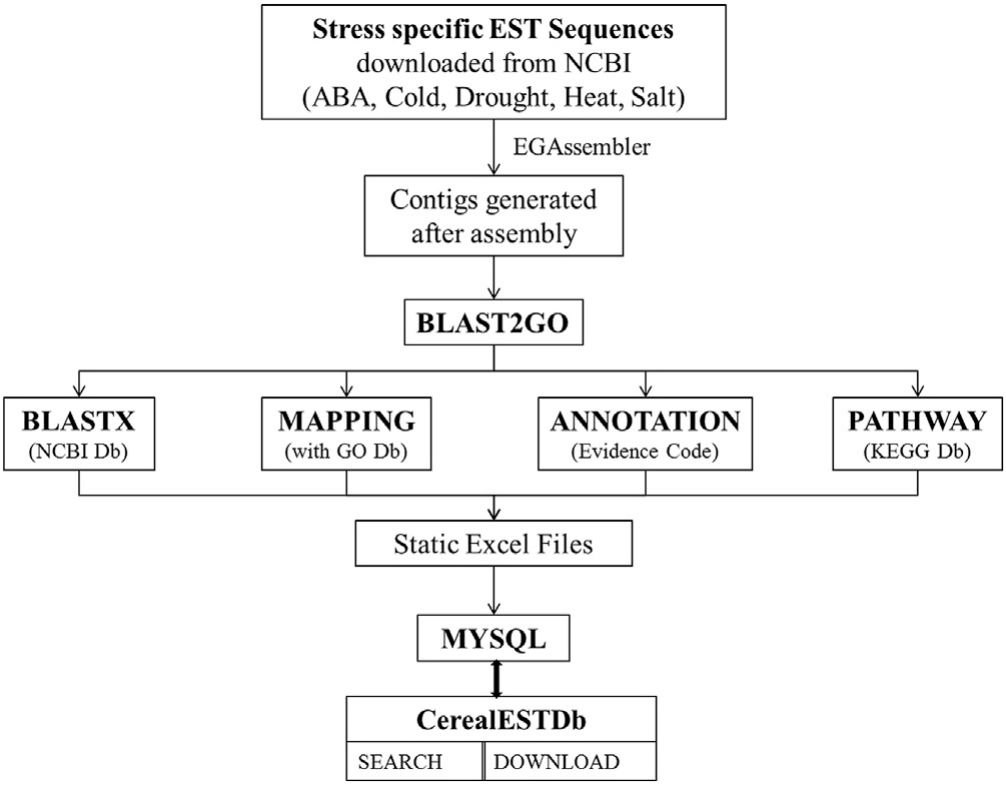
Schematic illustration of the process of development of CerealESTDb.

The EST sequences (ESTs) were searched and downloaded from National Centre for Biotechnology Information (NCBI) (www.ncbi.nlm.nih.gov) by using various combinations of terms crop name, the scientific name of crop, and stress name for four important cereal crops *viz*., maize, rice, sorghum, and wheat and five abiotic stresses *viz*., abscisic acid (ABA), cold, drought, heat, and salt stresses (Figure 1). These EST sequences were re-checked with the writing of suitable Perl scripts and filtered out all non-related EST sequences. The remaining EST sequences were also masked to eliminate the incorrect part in these sequences and then finally assembled using EGAssembler (https://www.genome.jp/tools/egassembler/) by considering the default parameter settings and the similarity-based clustering was set to 80 percent overlap identity (Masoudi-Nejad et al., 2006). These assembled EST-contigs were then translated to reading frames. The BLASTx algorithm was applied against the protein sequence database (NCBI nr) for the identification of potential translated products (Altschul et al., 1990). The homologous EST-contigs with annotated proteins that exists in the nr database were selected for functional annotations based on maximum E-value (1E^−3^) and the minimum alignment size of high scoring pair (HSP) length of 33. Blast2GO v 2.5 was employed to perform the functional analysis of the EST-contigs (Conesa and Gotz, 2008; Gotz et al., 2008). Further, the EST-contigs were categorized according to the GO vocabularies into three categories *i.e*. molecular function, biological process, and cellular component. These EST-contigs were mapped to the Kyoto Encyclopedia of Genes and Genomes (KEGG) database (Kanehisa and Goto, 2000) and attained the corresponding metabolic pathways. A flowchart depicting the pipeline for above-described process is presented in Figure 2.

### Software Architecture and Database Design

**FIGURE 3:**
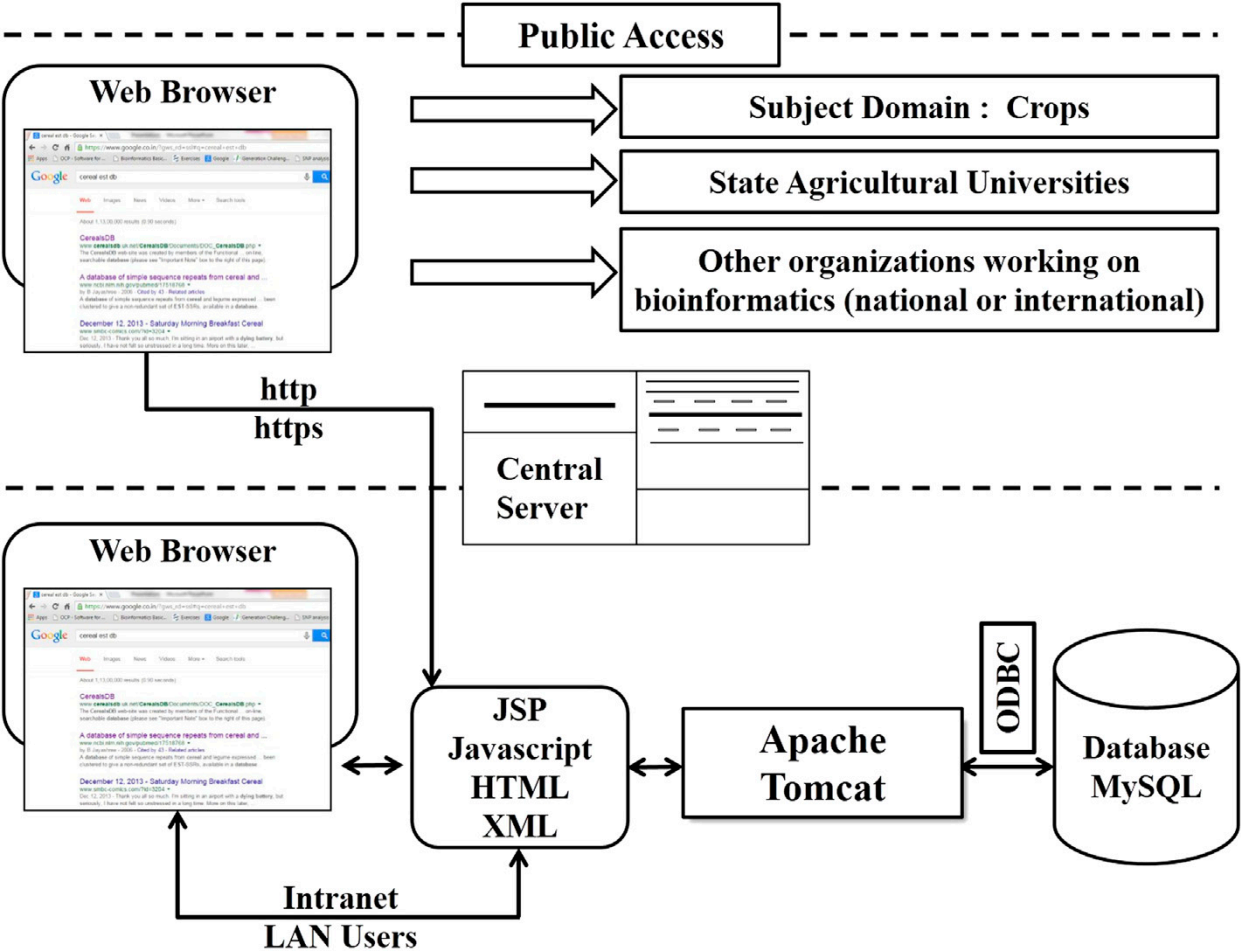
Three-tier architecture of the software used in CerealESTDb.

The CerealESTDb has been implemented using standard 3-tier web architecture (Lee et al., 2009) followed to develop this web application. Different components of CerealESTDb architecture are presented in Figure 3. Access to this database has been provided by developing an end-user interface using HyperText Markup Language (HTML), cascading style sheets (CSS), and javascript. The search request will be submitted to a web server i.e., apache-tomcat, and processed by corresponding servlets created using Java Server Pages (JSP) and javascript. These servlets fetched the data from the developed RDBMS in MySQL using Java Database Connectivity (JDBC) technology to process and send the response to the end user’s web browser.

The database has been implemented using MySQL and contained different information related to crop, stress, gene name, gene ontology, enzyme, and pathway. The design of the database has followed the normalization procedure of a relational database management system to maintain data consistency and avoid data redundancy in the database. The database comprises tables for crop, stress, contigs, gene ontology, enzyme, pathway, contigs_pathway, and pathway_enzyme. The entity-relationship (ER) diagram of this database is mentioned in Supplementary Figure S1.

### User Interface of CerealESTDb

The CerealESTDb comprises of Home, About, Search, Advance Search, Important Link, Contact Us, and Help. The “Home” page describes the database and its possible use in a molecular breeding program. The “About” page describes the process workflow used for extracting and processing the data, starting from collection of data to annotation of the genes and finally populating into the database. Various search options provide the facility to extract the stored information in two ways namely, “Search” (Supplementary Figure S2A) and “Advance Search” (Supplementary Figure S2B).

Several options have been provided in the web interface to enable a fast and efficient search for the end-user to retrieve the desired results with and meet the varied requirements of end users in terms of selecting various attributes. The users have the option to enter the free text in the search box or select the required values from dropdown lists. After selecting or entering the values for various input boxes, the users will click on the “Submit” button to obtain the required results. After clicking on the “Submit” button, the selected and entered values are converted into suitable Structured Query Language (SQL) format and fetched the results from the database and delivered the results in easy to use format to the end users’ browser window.

## Results and Discussion

**FIGURE 4:**
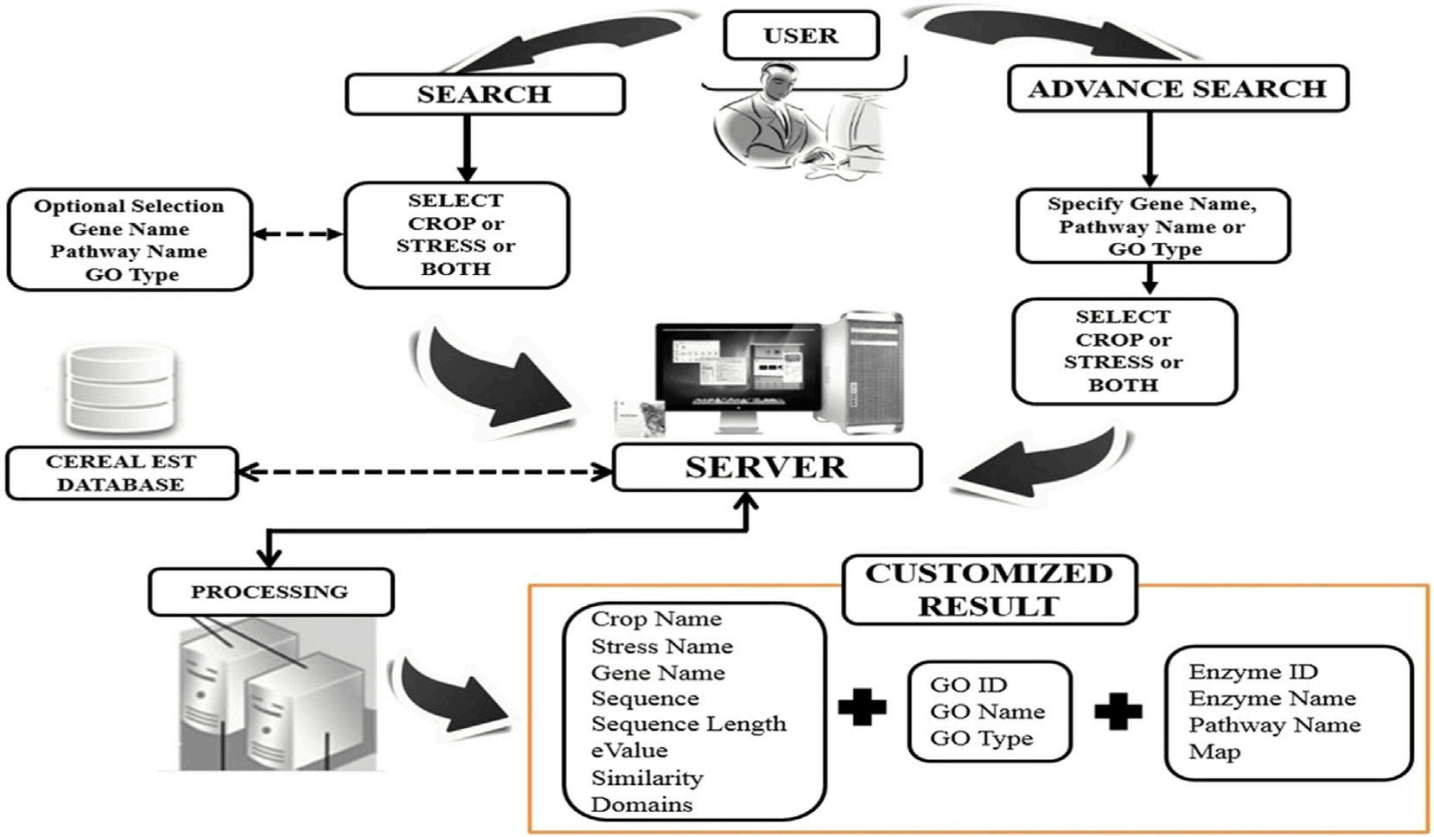
Browsing CerealESTDb features diagram.

Out of 610,893 ESTs downloaded for abiotic stresses viz., ABA, cold, drought, heat, and salt across all four crops viz., maize, rice, sorghum, and wheat, only 9% were assembled into contigs (55,826 in number). ESTs for cold stress in sorghum were not assembled because EGAssembler requires a minimum of 64 EST sequences and there were only 40 ESTs for cold stress in sorghum. There were a total of 51,791 genes that were annotated by BLAST out of 55,826 contigs across all the abiotic stresses. The total number of the assembled contigs and corresponding annotated BLAST genes against the *nr* database of NCBI, GO terms, and pathways are shown in Figure 1. The options to select the desired attributes have also been provided to the users and avoided the unnecessary attributes from the database that save the query response time too. Some of the important attribute names are selected by default and users have the option to either select or ignored other attributes, if needed. These results can further be combined with identified GO terms and associated pathways if any. The identified pathways will be helpful for retrieving the enzymatic information and hyperlink to pathways that can be further visualized for additional information or relationships. The users can also download the result in.xls format to the user’s desktop for later use. The above description is summarized in Figure 4 for a better understating of the retrieval process.

**FIGURE 5:**
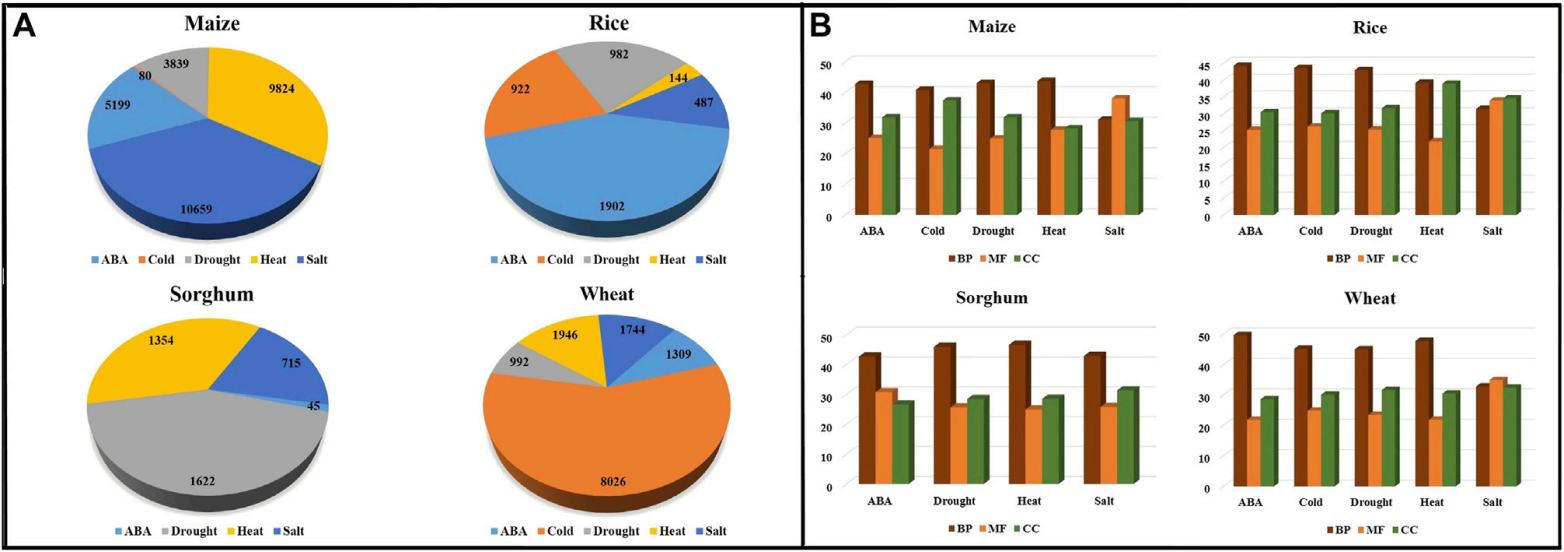
Distribution of **(A)** annotated contigs across different abiotic stresses in the crops. **(B)** GO terms—Biological Process (BP), Molecular Function (MF), and Cellular Component (CC) across abiotic stress in crops.

Stress-wise annotated genes are also represented using pie charts for the cereal crops under study (Figure 5A). Figure 5B shows the percentage of GO terms in different crops across these five abiotic stresses. The GO terms were further classified into three categories, biological process (BP), molecular function (MF), and cellular component (CC). The number of biological process-related GO terms are largest for each stress in all crops, followed by cellular component and molecular function, except in salt stress of maize, rice, and wheat.

Many plant genes have their expression modulated by stress conditions. The common genes in all crops and conditions were auxin-binding protein/auxin response factor (Guo et al., 2018), CCT motif transcription factor (Li and Xu, 2017), heat-shock proteins (Jacob et al., 2017; Shanker el at., 2012), Ser/Thr protein kinase (Islam et al., 2013), ribosomal proteins (Moin et al., 2017), and zinc-finger proteins (Zang et al., 2016). The small auxin upregulated RNA (SAUR) is a plant-specific gene family of auxin-responsive genes. As evident from previous reports, the overexpression of these SAUR genes contributed to the tolerance to drought and salt in *Arabidopsis* (Guo et al., 2018). Auxin-response factor proteins have been well studied in crop plants for their direct involvement in response to multiple stresses (Stortenbeker and Bemer, 2019). The heat-shock proteins play key roles in protecting plant cells or tissues from various abiotic stresses (Wen et al., 2017). Other common genes such as 14-3-3 genes, cytochrome c/b/p450, ubiquitin-like protein, etc., have been identified in the present study. The 14-3-3 genes are involved in various abiotic stresses of different plant species such as rice (Chen et al., 2006; Chen et al., 2019), *Populus* (Tian et al., 2015), Phaseolus vulgaris L. (Li et al., 2015), and *Hevea brasiliensis* (Yang et al., 2014), as reported by previous studies.

The maximum number of GO terms is pertaining to response to stress/defense response in all abiotic stresses for all considered plant species. Moreover, some GO terms are specific to the abiotic stress like Abscisic acid stimulus/signalling/recovery, drought response, response to heat, salt, and cold.

Further, there were 1,746 KEGG pathways associated with the annotated genes in all crops for these five stresses. The sugar metabolism pathway is the most common pathway in all cereal crops under study. Sugars are chemically active biomolecules and are involved in crucial Physico-chemical mechanisms such as photosynthesis, respiration, seed germination, flowering, senescence, and so forth. Therefore, modulating sugar composition or concentration in plants may improve their responses or adaptation to abiotic stress (Gangola and Ramadoss, 2018). The other common pathways were carbon fixation in photosynthetic organisms, purine metabolism, and metabolic pathways. Evidence suggests that certain purine metabolites like allantoin and allantoate, products of purine metabolism contribute to stress tolerance in plants (Casartelli et al., 2019; Watanabe et al., 2014).

**FIGURE 6:**
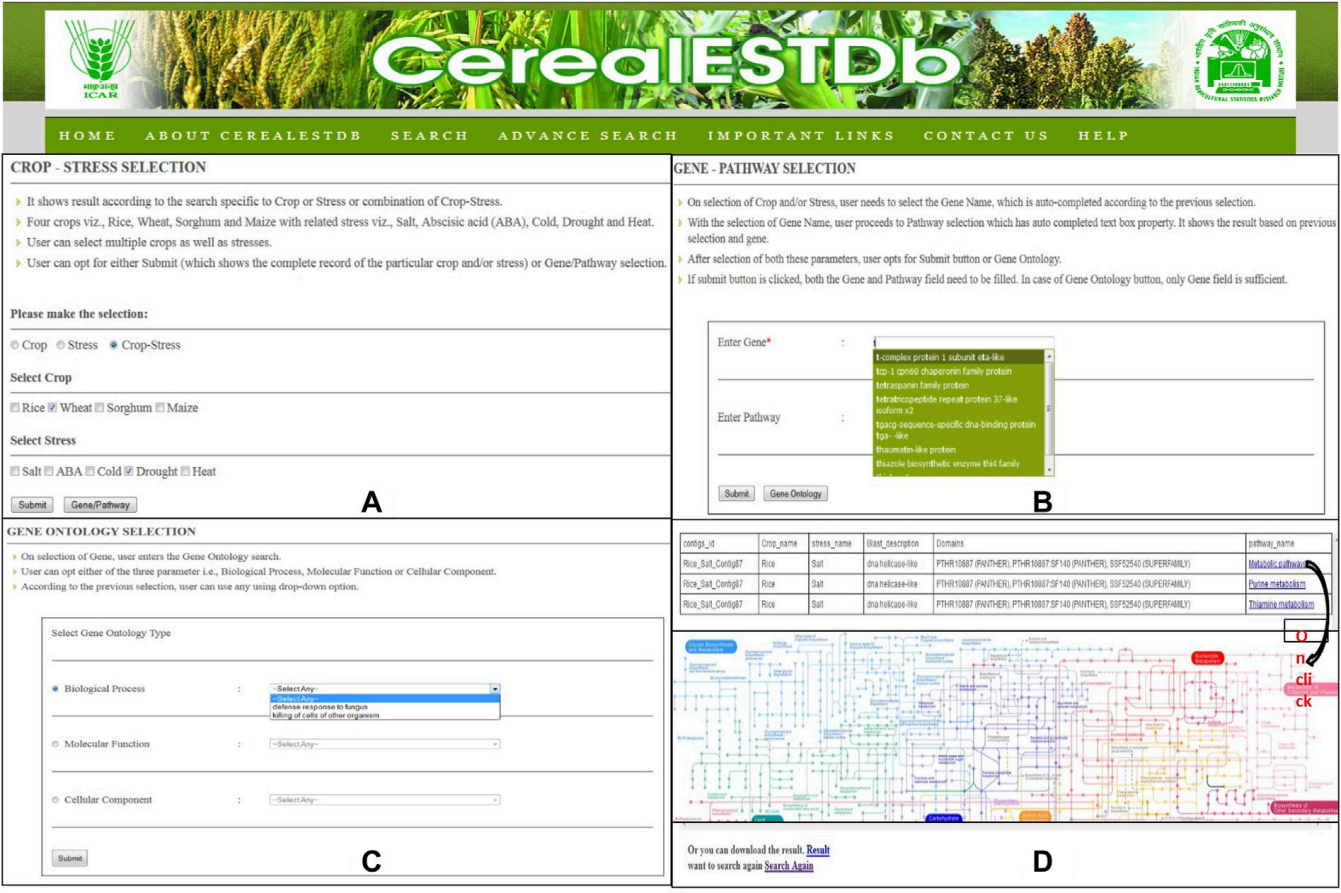
Details of Search Options in CerealESTDb **(A)** Crop-Stress Selection page with options of select either crop or stress or combination of both **(B)** Gene-Pathway Selection page with mandatory field Gene and optional field Pathway **(C)** Gene Ontology Selection page, to select either of the three options of Biological Process or Molecular Function or Cellular Component **(D)** Final Result page after going through all the three selection pages with pathway image (on click).

The user interface of this database has been developed to provide access to the compiled resources in the form of CerealESTDb and important links have been made for Home, About CerealESTDb, Search, Advance Search, Important Links, Contact Us, and Help. There are two types of search options developed in the user interface in the form of “Search” and “Advanced Search”. The first option allows the user to select crop or stress or a combination of crop and associated stress (Figure 6A).

The users have the option to either click on “Submit” or “Genes/Pathway”. If a user clicks on “Submit,” it will further ask users to select the list of attributes or parameters from available attributes (i.e., Crop Name, Stress Name, Gene Name, Sequence, Sequence length, e-value, similarity, and domains). After selecting the desired attributes, the required information will be displayed to the users and this information can also be downloaded and exported in.xls format for further analysis. Another option is to select genes and corresponding pathways for selected Crop/Stress/Crop-Stress and further choose the required attributes for additional information of the selected combination (Figure 6B). After selecting the gene/pathway option, it is compulsory to enter the gene name to proceed to the next level of search to retrieve the desired results.

Further, the users have the option to search by specifying “Gene Ontology” terms related to biological process, molecular function, and cellular component (Figure 6C). The results under each selection will be displayed to the end-user along with a pathway map (Figure 6D). The user can download these results in.xls format and the corresponding pathway map can also be saved as.gif.

**FIGURE 7:**
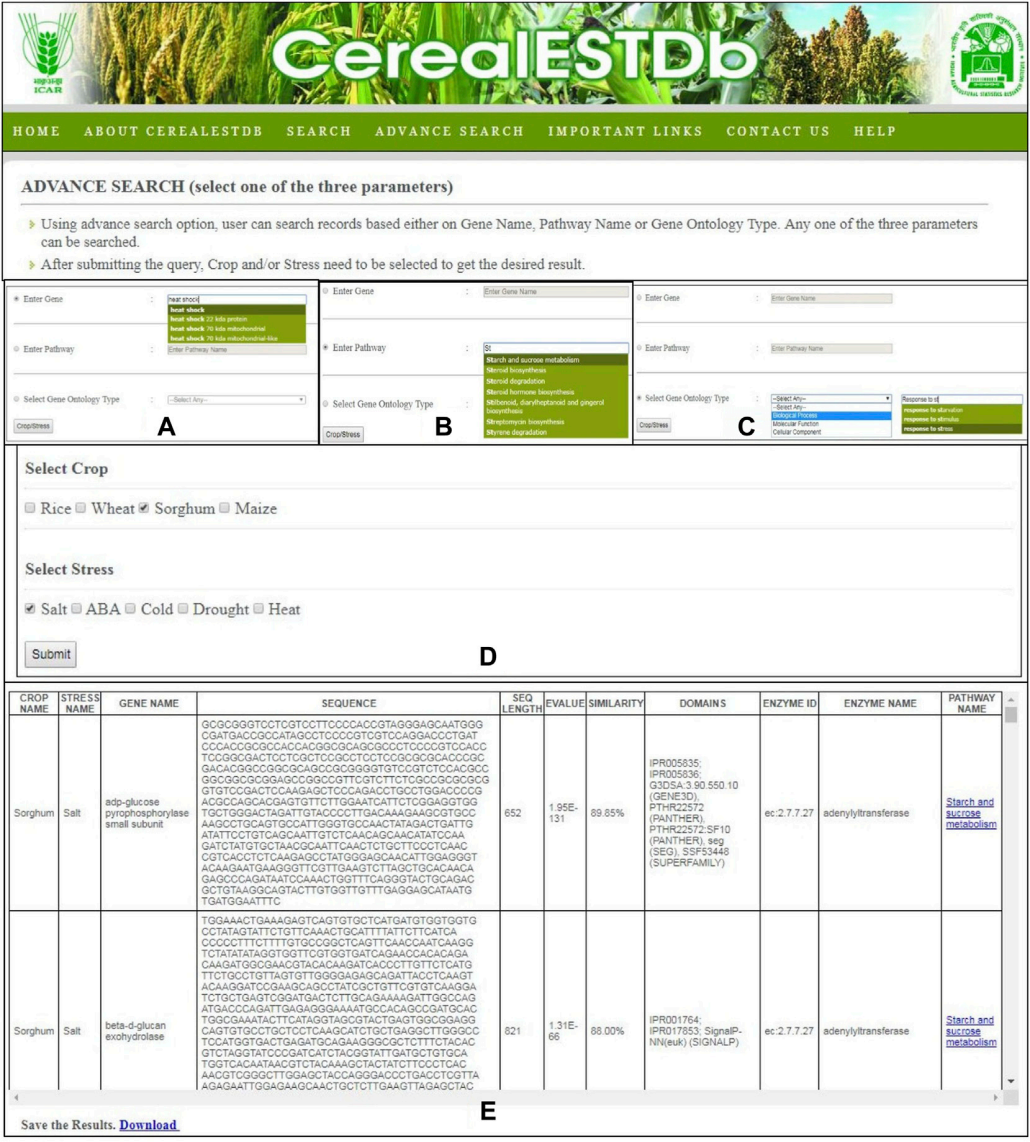
Details of Advance Search options of CerealESTDb. The search could be done through three options **(A)** Gene name search or **(B)** Pathway name search or **(C)** Gene ontology term search, to select either of the three options of Biological Process or Molecular Function or Cellular Component and their corresponding values **(D)** Common Crop-Stress Selection page with options of select either crop or stress or combination of both **(E)** Final Result page after selecting either of the three options.

The “Advanced Search” option provides users the ability to directly search information from this searchable database by selecting a specific gene name (Figure 7A), pathway name (Figure 7B), or gene ontology type (Figure 7C). The user is then asked to select the combination of crop name and stress names (Figure 7D). The relevant tabular information is displayed to the user which can be downloaded in.xls format (Figure 7E). In order to make the search user-friendly, the gene name, pathway name, and ontology type will be selected through a drop-down menu displayed from the database to the user after typing the first few letters of these in the search options.

“Important Link” helps the user find the web pages of relevant organizations and databases. “Contact Us” gives contact details of research team members and “Help” provides steps and flow of various search routes of this database.

The features of the CerealESTDb are:

a. User-friendly, searchable, and interactive data resource for searching a set of genomic information associated with response to abiotic stresses in cereal crops.
b. Provides a unique platform for interactive retrieval of annotated genes, GO terms, and corresponding generated metabolic pathways.
c. Provides alternate ways to explore 51,791 genes, 254,609 GO terms, and 1,746 generated metabolic pathways
d. Provides a facility to users to export results in.xls format for further annotations.

The few advantages of the present database are given below:

a. The developed database will provide the desired information related to search queries even without knowing anything specific to search query criteria and also facilitate the users through selection.
b. In this database, an effort has been made to collect various ESTs related to four important cereal crops across five specific abiotic stresses. A pipeline has been developed to mine the genes/proteins and that can also be applied in other plant species to annotate the ESTs and gene models.
c. The present database provides extensive information on metabolic pathways and extracted GO information with the help of BLAST2Go tool specific to particular abiotic stress i.e. ABA, cold, drought, heat, or salt-related to considered cereal crops.

## Conclusion

The CerealESTdb, a user-friendly, searchable, and interactive database has been developed with a primary goal to provide information on assembled and annotated ESTs from four major crop plants, namely wheat, rice, maize, and sorghum under multiple environmental stresses including cold, heat, drought, and salt stress as well as on the application of ABA. This cohesive database will also provide extensive information about genes, ontology, and corresponding metabolic pathways related to these ESTs. The CerealESTdb also facilitates users to access a description of the biological functions of genes involved in more than one related abiotic stress. This database will help in providing new solutions to molecular biologists and plant breeders for accelerating their efforts to develop abiotic stress-resistant cultivars without compromising the nutritional quality through introgression and gene pyramiding. With further progress in NGS methods and the availability of genome sequences data in the public domain, we hope that statistics of CerealESTdb will eventually grow by two-to-three folds in upcoming years.

## Data Availability

The original contributions presented in the study are included in the article/Supplementary Materials, further inquiries can be directed to the corresponding author.

## Supplementary Material

Supplementary Figure S1 Entity-Relationship Diagram

Supplementary Figure S2 Step-wise options followed in **(A)** simple Search page and **(B)** Advance Search page
