# Supplementary figures and images for: CerealESTDb: A Comprehensive Resource for Abiotic Stress-Responsive Annotated ESTs With Predicted Genes, Gene Ontology, and Metabolic Pathways in Major Cereal Crops
Source: Front Genet. 2022 Feb 24;13:842868. doi: 10.3389/fgene.2022.842868 (PMC8907976; doi:10.3389/fgene.2022.842868)

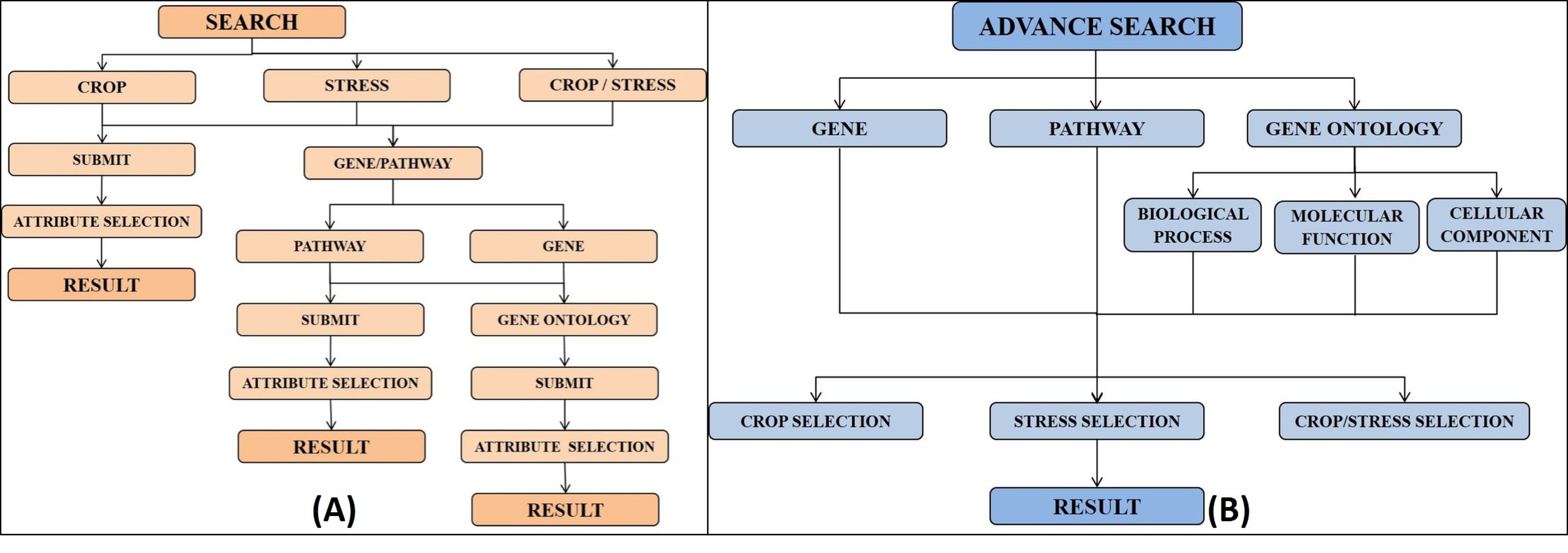

Supplement: Supplementary file 1 [file Image2.jpg]

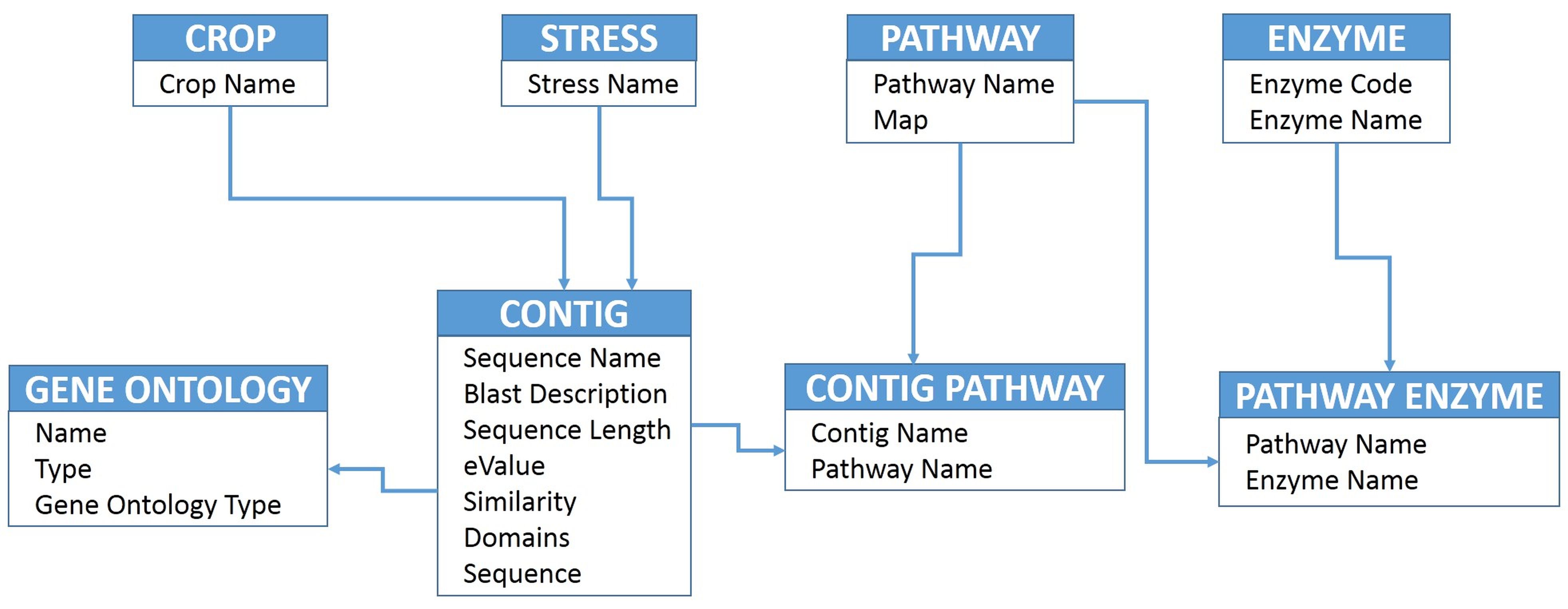

Supplement: Supplementary file 2 [file Image1.jpg]
